# Knowledge sharing in virtual communities of practice of family caregivers of people with Alzheimer’s

**DOI:** 10.1186/s12877-024-05045-7

**Published:** 2024-07-04

**Authors:** M. Romero-Mas, A. M. Cox, A. Ramon-Aribau, Beni Gómez-Zúñiga

**Affiliations:** 1 Health Sciences and Welfare Faculty, University of Vic-Central University of Catalonia, Vic (Barcelona), Spain; 2 Information School, University of Sheffield, Sheffield, UK; 3 Estudis de Psicologia i Ciències de l’Educació, Universitat Oberta de Catalunya, Rambla Poblenou, 156, Barcelona, 08018 Spain

**Keywords:** Knowledge sharing, Family Caregiver, Alzheimer’s Disease, Shared Community, Mobile Application

## Abstract

**Introduction:**

Knowledge sharing can only happen in the context of a trusting and supportive environment, such as evolves in communities of practice and their virtual equivalent, virtual communities of practice. The main objective of this study was to understand knowledge sharing between participants in a virtual community of practice of caregivers of people with Alzheimer’s.

**Methods:**

The authors designed their own mobile application, and two virtual communities of practice were created independently and differentiated by how they were moderated: one by an expert caregiver and the other by three health professionals. 38 caregivers and four moderators were involved in the study, which ran between July 2017 and April 2018. A total of 1925 messages were exchanged within the two communities and used as data in the study. Message data was analysed using LINKS (Leveraging Internet Networks for knowledge sharing).

**Results:**

Participants were more motivated to acquire knowledge related to caring for the person with Alzheimer’s rather than caring for themselves. The purpose of the messages was to inform others about the sender and not to seek answers. It seems that the interaction was more to socialise and to feel heard, than to gain information. Face to face meetings appear to have accelerated community development. On nearly every parameter, behaviour was significantly different in the two communities, reflecting the importance of the character of the moderator. Caring for oneself was a much stronger theme in the community that included health professionals. Experiential knowledge sharing was particularly strong in the group led by a caregiver.

**Discussion:**

Caregivers adapted the virtual community of practice to their own needs and mainly shared social knowledge. This focus on social support, which seems to be more valued by the caregivers than information about the disease, was not an expected pattern. Virtual communities of practice where peers count on each other, function more as a support group, whereas those moderated by health professionals function more as a place to go to acquire information. The level of interactivity points to such communities being important for knowledge sharing not mere knowledge transfer.

## Introduction

Knowledge sharing, as a central knowledge management practice, can be regarded as “an exchange of knowledge between people as members of a community or an organisation” [1]. In knowledge sharing, there is an emphasis on interactivity; it implies interaction between two parties, unlike knowledge transfer which is just a one-way process of someone giving something to someone else. It follows that knowledge sharing can only happen in the context of a trusting and supportive environment.

Communities of practice are highly valued as they have been identified as effective loci for the creation and sharing of knowledge [2]. The community of practice concept captures the way that individuals share knowledge, their desire to learn and support each other, and it is rooted in common daily practices and a common identity [3]. Among the main reasons why communities of practice are effective tools for knowledge sharing is the fact that much of an individual’s knowledge is intangible and tacit in character [4]. Communities of practice offer the means for sharing this experiential knowledge because the development of a strong network of likeminded-individuals who share a common understanding is an environment characterised by trust, mutual respect, shared behavioural norms and reciprocity [5].

Communities of practice have developed an important role in the healthcare field. This is because healthcare knowledge sharing, which is “the explication and dissemination of context-sensitive healthcare knowledge by and for healthcare stakeholders through a collaborative communication medium in order to advance the knowledge quotient of the participating healthcare stakeholders” [6], is essential in healthcare organisations.

Communities of practice often take the form of virtual communities of practice and they have gained high acceptance by people as places to learn and share knowledge [7]. Virtual communities afford a new knowledge exchange model where knowledge sharing activities are considered a type of social exchange behaviour [8]. The main benefits of health virtual communities of practice are increased interaction among the members, the processes of creating and sharing knowledge, peer, social and emotional support, public health surveillance and the potential to influence health policy [9]. However, few studies can be found which tackle knowledge sharing between family caregivers (hereafter, ‘caregivers’) [10, 11].

Over the past decades, life expectancy has increased considerably, making the challenges posed by chronic age-related conditions more difficult. One of these disabling chronic disorders that requires a high level of care is dementia. 47 million people worldwide live with dementia, and this statistic is expected to increase to 75 million by 2030 and to 132 million by 2050 [12]. 80% of dementia cases are the result of Alzheimer’s disease [13]. However, Alzheimer’s not only affects those who suffer from it, but also those around them. Carers often suffer from, for example, physical, psychological or economic problems, due the maintenance of their relative. In this context, the role of family caregivers is of major importance as they become the invisible second patients [14]. Caregivers are individuals who have a significant personal relationship with, and provide a broad range of unpaid assistance to, an older person or an adult with a chronic or disabling condition outside of a professional or formal framework [15]. Due to the growth of dementia and Alzheimer’s, caregivers have a key role in the health system. Their importance may be seen in the example of Spain, where the health system only covers 20% of the total time devoted to long-term care and the remaining 80% is performed within the informal care system [16].

Despite this, most healthcare delivery models focus primarily on individual patients and do not properly engage, educate, or support caregivers [17, 18]. In addition, web-based interventions aimed at supporting caregivers of people living with dementia may improve their psychological health [19] and their participation in virtual communities has been associated with reducing their caregiving burden [20]. Information technologies, therefore, have the potential to support the shift of the healthcare paradigm to a more user-centered approach and, even to a family and caregiver-centered approach [21].

Caregivers have a lack of skills and training for the job they are required to do and they have to cope with this role in addition to continuing their daily lives [22]. They need to: learn skills for improving the daily life management of their relative [23], have access to information [24], look after their own personal health and receive help from others [24] and gain support and anticipatory guidance [25], especially from others in the same situation [15]. Furthermore, they need social support as it provides a means for them to share their caregiving experience, build social relationships and receive emotional comfort and informational material [26] ). The virtual community of practice constructed through the knowledge sharing process may help caregivers to address these needs.

Caregivers can feel better when they share their experiences and learn that there are other people facing the same kinds of problems [27]. However, caregivers say that the weaknesses in support from general practitioners is chiefly due to a lack of information from advice and assistance services (55%). General practitioners frequently fail to identify or involve caregivers early enough in the treatment of patients (42%) [28]. What ads to the complexity is the fact that Alzheimer’s is a chronic illness with several stages and not all caregivers share the same circumstances. Hence, practitioners should realise that caregivers may have different information and support needs and that those needs may change throughout the caregiving experience [29].

This paper studies the knowledge sharing by caregivers in virtual communities of practice adopting the model called LINKS, which is grounded in the community of practice concept [6], and which has been used for similar studies [30, 31]. The authors of this model developed it, arguing that to generate holistic healthcare knowledge it is important to back up explicit knowledge with experiential knowledge [31]. Explicit knowledge is codified knowledge represented by information in journals, clinical pathways, protocols, and procedures [32], and it describes how things should work. Experiential or tacit knowledge is the non-formalised knowledge of participants and embodies their experiential know-how, skills, and intuitive judgement about what really works and how to make it work [6]. When considering caregivers’ needs and the characteristics of virtual communities of practice, we expect that social support and social knowledge will be predominant in this knowledge-sharing research.

The LINKS model characterises healthcare knowledge sharing solutions at three interrelated levels: conceptual, operational, and compliance [6]. The conceptual level has three dimensions for knowledge sharing: medium (the digital media channels used), context (topics and motivations for knowledge sharing) and modalities (type of knowledge shared). The operational level of the model highlights the culture of collaboration among knowledge stakeholders from varying backgrounds and with varying roles. The model addresses the trust the users have in online communities at the compliance level [33]. In this study, we focus on the conceptual level as most relevant to knowledge sharing.

The average duration of Alzheimer’s is 8 to 10 years. In the course of this period, the patients’ health by stages so their caregivers need to learn from experience the skills to care for them. Furthermore, literature shows that an unmet need is a lack of quality information about support strategies or services that can help to alleviate the challenges for the caregiver [34]. “Quality information” in this context would be represented by information, guidance, or consultation that is effectively tailored to meet the needs of individual dementia caregivers in ways that are helpful and feasible. In this research, we use a virtual community of practice for caregivers with people with Alzheimer’s as a mechanism for peer-to-peer knowledge sharing to support the acquisition of evolving knowledge needs. In these communities, a moderator is the coordinator who plays an integral role in enhancing the functioning of the community and facilitating learning [35]. That moderator will have the role of facilitator of the sharing of information, help to activate knowledge sharing and give support to all the members, within both the caring context and the technological one [34, 36]. In our case, expecting different behaviours of the communities depending on the character of the moderator, two virtual communities of practice were established and developed with different profiles of moderators: one with an expert caregiver and the other with three health professionals.

Hence, the main objective of the study is to gain a deeper understanding of the knowledge sharing between participants in a virtual community of practice of caregivers using the LINKS model. What is the role of knowledge sharing in virtual communities of practice for caregivers? Is it a significant variable in these communities? As the caregivers were not part of any virtual caregiver community, a specific application was developed to create these virtual communities of practice. Therefore, to enhance comprehension of knowledge sharing among participants, we also analysed the participants’ activity within these communities. Firstly, we specifically analysed the medium of knowledge sharing. Then, we tackled the knowledge sharing context. After that, the modality of knowledge sharing between caregivers was examined. Finally, we investigated the characteristics of the virtual community of practice by analysing the impact of having different types of moderators.

## Materials and methods

### Study design and procedure

The lack of generally available tools and resources to establish virtual communities of practice [37] prompted the researchers to design their own mobile application (app). In this way, access to the interactions made by the participants was gained. And thus, they could be analysed to investigate knowledge sharing. The app “I’mWithYou” was designed following the virtual community of practice framework for designing virtual communities of practice of caregivers, focusing on eleven dimensions [38]. Hence, the medium chosen to share the knowledge between participants was the “I’mWithYou” app.

Two virtual community of practice were created independently: one with an expert caregiver as the moderator and the other with three health professionals as the moderators. Our independent variable (type of moderator) has two conditions: one must be led by an expert caregiver, the other by health professionals. We assigned participants randomly to each experimental group. That gave us our independent group design.

The app included a wall for discussion where a thread could be initiated by a text, a photo, an event, or a poll. Each thread had a brief descriptor of the message labelled (“disease”, “caring for others” and “caring for oneself”). To follow a thread, members could “comment” or “like” the message. There was also a direct messages option (no label could be assigned to these messages).

### Participants

All participants were recruited between July 2017 and April 2018. 38 caregivers and three health professionals were involved. The communities were active from the 24th of April 2018 to the 20th of February 2019. In order to recruit the caregivers for the study, researchers first shared the project idea with the Association of Alzheimer’s Family Caregivers (AFMADO) in the region of Osona, Catalonia (Spain). It was then disseminated to the hospital healthcare system and community health and social fields throughout Osona. In total, five explanatory sessions with caregivers (individual and group) and twelve sessions with health professionals (individual and group) were held. From these sessions, the researchers were able to recruit 38 caregivers and 3 health professionals.

Access to the app was exclusively for participants. The inclusion criteria were that participants should be a caregiver of people with Alzheimer’s, have Internet access, be able to use a smartphone, tablet or computer, and have their relative living in Osona. The exclusion criteria were caregivers who did not want to participate, and caregivers who did not have an email address.

The inclusion criteria for the health professionals were the following: being a professional in health and/or social care; working with people with Alzheimer’s and/or caregivers; having Internet access; being skilled in using a smartphone, tablet or computer; and living in Osona. Health professionals who had no email or were not eager to participate in the study were ruled out.

The caregivers in both virtual communities of practice were mainly married females, between 55 and 57 years of age, with one or two children and with an education up to secondary studies. On average, they had been carers of their own parents with Alzheimer’s for two years. As no significant statistical differences were found between them, we could proceed to compare them.

The role of moderators in virtual communities of practice is crucial since they are requested to foster a vibrant community where knowledge exchange thrives [35]. In this case, the main goal was to compare the presence/absence of health professionals. In the “I’mWithYou” virtual community of practice, the moderator was an expert caregiver who had been taking care of her spouse for 8 years and fulfilled the same inclusion criteria as all the other caregivers. She initiated 25.5% of the conversations in her community. Whereas the “I’mWithYouPlus” virtual community of practice included three health professionals (a nurse, a psychologist and a geriatric physician), all with more than 10 years of experience working with people with Alzheimer’s and their informal caregivers. They initiated 49.5% of the conversations in their community.

Three face-to-face sessions were facilitated for the members. The initial meeting was timed when the app became available for download. This meeting primarily assessed the download process and usability of the app, along with participant introductions. The objectives of the second meeting included socialization, engaging in discussions that would continue in the virtual forum, and identifying challenges that could potentially imped community efforts. The final in-person meeting focused on evaluation, dissemination, and concluding activities related to the app. All moderators were invited to attend the three face-to-face meetings with all the regular participants, and their role in these sessions was like that of any other participant. In addition, moderators attended three more meetings alone with the researchers, in order to address their doubts, to remind them of the virtual community of practice goals, to motivate them and encourage their activity in the virtual communities of practice. Administrative and technological aspects were handled by the researchers.

### Data collection

All messages were registered and stored separately on the communities’ web server as the literature points out that moderators are key when considering the knowledge of a virtual community of practice [7], the main goal of having two virtual communities of practice was to compare the presence/absence of health professionals. All messages could be retrieved and tabulated: activity identifier (activity number), sub-activity identifier (activity number in case of comment or like), type (text, a photo, an event or a poll), label (“disease”, “caring for others” and “caring for oneself”), day, time, content (the message itself), user identifier, and recipient identifier. A total of 1925 messages were exchanged within the two communities: 933 (“I’mWithYou”) and 992 (“I’mWithYouPlus”). From these messages, a total of 552 were posted by moderators (163 in the “I’mWithYou” and 389 in the “I’mWithYouPlus”).

### Data analysis

Data was analysed following the LINKS model [6], focusing on the conceptual level, which has the three dimensions of knowledge sharing: medium, context and modality.

The authors decided that the LINKS model was suitable for analysing the knowledge sharing in the virtual community of practice of caregivers, as previous studies already followed this model to examine the knowledge sharing in a health virtual community of practice [30, 31]. The purpose was to use the LINKS model to understand and compare the knowledge shared in two virtual communities of practice.

The researchers proposed a deductive categorisation of messages which may be seen in Table 1:

**Table 1:** Categorisation at the Conceptual level

Type of Knowledge	Categories	Variables
Knowledge medium	Message type	Post (text, photo, poll, event)
		Comment
		Like
		Direct Message
	Label	Disease
		Caring for others
		Caring for oneself
	Purpose	Information (about disease, about sender, about app, Internet Link)
		Interactivity (seeks answer, does not seek answer)
Knowledge sharing context	Topic	Social support (give support, seek support, gratitude)
		Skills to care for people with Alzheimer’s
		Disease information
		Caring for oneself
		About the research
	Temporal relevance	Time of day (dawn, morning, noon, afternoon, evening, night)
		Month of the intervention (M1-M11)
Health knowledge modality	Modality	Explicit
		Experiential
		Social

The researchers could directly categorise the type and label of the knowledge sharing medium, and temporal relevance of the knowledge sharing context. A content analysis permitted us to complete the rest of the categories: purpose of the knowledge sharing medium, topics shared according to the caregivers’ needs of the knowledge sharing context and knowledge sharing modality. Motivation was the only category of knowledge sharing context which was not possible to study in this research.

A total of 1695 messages were posted. “I’mWithYou” posted 865 messages (18.84% by moderator) whereas “I’mWithYouPlus” posted 839 (46.36% by moderators). All messages were coded by two researchers, “like” messages having been removed. The consistency of coding was checked through inter-coder reliability checking between the two researchers, and the level of agreement achieved was of 0.787 Kappa, coefficient accepted as 0.75%.

The knowledge sharing medium relates to the purpose for which the participant exchanges knowledge and was classified following the “information” and “interactivity” constructs [39]. “Information” messages were the ones whose primary purpose was solely to inform and to provide relevant information to participants. “Interactivity” included the messages which promoted dialogic relationship-building. These two constructs where subcategorised: “information” (“about disease”, “sender”, “app” or “Internet link”) and “interactivity” (“seeks answer”, or “does not”).

The knowledge sharing context is caring for people with Alzheimer’s and we included two elements: a specific topic to classify the theme of the knowledge sharing and temporal relevance (timing of the knowledge sharing with respect to the caring). The specific topics identified from the caregivers’ needs were: “social support” (give or seek support, gratitude), “skills to care for people with Alzheimer’s”, “disease information”, “caring for oneself” and “about the research”. “Skills to care for people with Alzheimer’s” is when the content is directly related to the people with Alzheimer’s [23]. “Disease information” is information about Alzheimer’s. “Caring for oneself” is any activity that was deliberately performed in order to take care of the caregiver’s own mental, emotional and physical health [40]. Finally, messages were categorised as “about the research” when users shared information about the app and/or how the study itself worked.

The health knowledge modality is focused on the types of knowledge sharing. We assigned three categories which were not mutually exclusive: “explicit”, “experiential” and “social”. We hoped that participants would share experiences and intuitive judgement about what really works in care and how to make it work. Furthermore, we expected that the virtual communities of practice would be helpful sharing explicit knowledge, increase experiential knowledge and help caregivers socialise.

SPSS 23.0 was used for all data analysis of the coded data.

### Ethics

Participants signed an informed consent form. Ethics approval was obtained from the University of Vic-Central University of Catalonia Ethics Committee (Reference 15/2017).

## Results

This section will be organised following the LINKS model shown in Table 1.

### Knowledge medium

In the case of the message type, “Comment” was the most common message in both groups, while “event” and “poll” were hardly used. The two communities mainly shared “text”, and they interacted using “like”, “comment” and “direct message”. Nevertheless, “I’mWithYou” shared many more “photo” whereas “I’mWithYouPlus” sent many more “direct messages”. The Chi-Square test suggests that there are significant statistical differences between the two virtual communities of practice with regards to the message type (< 0.001).

As for labels, all messages apart from “direct message” were labelled by the participants at the time of writing. Participants mostly labelled their messages as “caring for others”. However, “I’mWithYou” prioritised the disease whereas “I’mWithYouPlus” gave more importance to caring for yourself. The Chi-Square test suggests that there are significant statistical differences between the two VCoPs with regards to the “label” messages (< 0.001). Finally, regarding the purpose, in the two communities, the principal purpose of the message was” informative messaging about the sender”. Nevertheless, “I’mWithYouPlus” shared a very similar quantity of messages regarding the “disease”. Moreover, most of the messages did “not seek answers” as they did not explicitly ask questions. It seems that the interaction was more to socialise and to feel heard. The Chi-Square tests suggested that there are statistically significant differences in all the purpose subcategories “giving information” (< 0.001), apart from “interactivity” (0.162).

### Knowledge sharing context

At this point, the “like” messages were removed (they do not include content). “Direct messages” were again included. In total, there were 856 messages in the “I’mWithYou” virtual community of practice and 839 in the “I’mWithYouPlus” virtual community of practice. Regarding the temporal relevance, members could participate at any time during the day, although the most active participation was either in the “morning” or “afternoon”. The daily message average was 3.1 in “I’mWithYou” and 3.2 in “I’mWithYouPlus”. Nevertheless, the length of the periods of activity was different. Hence, in “I’mWithYou” the busiest period per hour was “evening”, with an even spread throughought day. In “I’mWithYouPlus” the busiest period was “evening”, followed by “afternoon”. Monthly, the mean was 56.54 messages in the “I’mWithYou” virtual community of practice and 70.91 in the “I’mWithYouPlus” virtual community of practice. The face-to-face meetings took place in “M1” (Month 1), “M8” and “M11”, and the moderators’ meetings in “M2”, “M3” and “M6”. Therefore, the face-to-face meetings characterised the virtual community of practice development as there was a positive impact in terms of number of messages. The virtual community of practice with professionals took longer to develop. Even though the two communities were highly active in the second month, it was not until month 7 and later that the virtual community of practice with professionals reflected the most activity.

Continuing with the knowledge sharing context, regarding the topic, the most important topic for both groups was “social support”. Participants were mainly “giving support”. Despite this, the virtual community of practice with professionals “prioritised gratitude” rather than “seeking support” and it showed more interest in “disease information” than in “skills in relation with the people with Alzheimer’s”. The Chi-Square test suggested that there were significant statistical differences between the two virtual communities of practice with regards to all the variables of the knowledge sharing context (< 0.001) apart from “about research” (0.201).

### Health knowledge modality

The main knowledge sharing modality was “social”. Most of the participants wanted to socialise within the group. The second most shared modality was “explicit”. However, “I’mWithYouPlus” virtual community of practice shared a similar quantity of “explicit” messages as “experiential” ones. The Chi-Square test suggested that there are statistically significant differences in all the variables of the knowledge shared modality (“explicit” <0.001, “experiential” =0.014 and “social”= 0.004).

To summarise, the types of messages, the labels used, and the purpose of knowledge sharing were statistically significantly different in the two communities. The unlike characteristics between these two communities make knowledge sharing different. Interactivity as a purpose does not depend on the type of community. The way the topics are used within the knowledge sharing context is also statistically significant, which means that the different characteristics of the communities cause the topics to be used differently, except for the “about research” topic. Finally, the differences in the health knowledge modality are also significant, so the type of community will determine the modality. The fact that there are statistically significant differences in most forms of knowledge sharing in the LINKS model means that the composition of these two communities, one with an expert caregiver and the other with health professionals, influences the knowledge sharing medium, context and the modality.

At summary of the main differences between the virtual community of practice including health professionals or not, may be seen in Table 2:

**Table 2:** Differences between the two virtual communities of practice in knowledge sharing activity

Type of Knowledge	Variable	“I’mWithYou” Led by an experienced carer	“I’mWithYouPlus” Led by health professionals
**Knowledge sharing medium**	**Message**	-High quantity of “comment”. -Considerable quantity of “photos”.	-High quantity of “like” messages. -High number of “direct message”.
	**Label**	-Priority for “disease” over “caring for yourself”.	-Priority for “caring for yourself” over “disease”.
	**Purpose**	-Extensive informative messages “about sender”. -More informative messages “about the app” than providing “an Internet link”.	-Informative messages quite split between “about sender” and “about disease”. -More informative messages providing “an Internet link” than “about the app”.
**Knowledge sharing context**	**Time**	-Messages are sent in the “morning”. -Most messages in “M3”, “M6”, “M8”: quicker community development.	-Messages mostly sent in the “afternoon”. -Most messages in “M2”, “M8”, “M9”: slower community development.
	**Topic**	-After “social support”, the second topic is “skills in relation to the person with Alzheimer’s” before “disease information”. -Priority for “seeks support” over “gratitude”. -Last priority for “caring for yourself”.	-After “social support”, the second topic is “disease information” before “skills in relation to the person with Alzheimer’s”. -Priority for “gratitude” over “seeks support”. - Last priority for “about of the research”.
**Knowledge sharing modality**	**Modality**	-Few messages of “explicit” knowledge.	-Similar numbers of messages regarding “explicit” and “experiential”.

## Discussion

Results showed differences in nearly all the categories of analysis which indicates that having different profiles of moderators in the virtual communities of practice is an important factor. In relation to the knowledge sharing medium, the virtual community of practice without health professionals tended to socialise as they shared a lot of photos. The other virtual community of practice used more direct messages which might be due to personal counselling from the health practitioners. Even though the influence of the health professionals counselling, the caregivers of the “I’mWithYouPlus” virtual community of practice labelled more messages with “caring for oneself” than with “disease”. The impact of “caring for oneself” in “I’mWithYouPlus” was the double than in “I’mWithYou”. Here it can be seen that health professionals were concerned about the health of caregivers as they suffer from a burden, and they can experience deterioration in their quality of life [41] ). Therefore, they insisted on the fact that caregivers need to take care of themselves as they have the tendency to prioritise the care-recipient. This is perhaps the opposite of what one might expect as health professionals have extensive knowledge of the disease and the literature indicates that caregivers would like to receive more information and support from the health professionals [28]. Having said this, the influence of the health professionals in the “I’mWithYouPlus” is reflected in the purpose of the informative messages as in this community they shared a similar number of messages about themselves and about the disease.

In the health professionals led community explicit knowledge was also shared to a significant degree, though at a much lower level than social knowledge. This reflects the priorities of practitioners to impart useful health knowledge. In the other group, after social knowledge, the main knowledge sharing was experiential knowledge about how in practice to care for the person being cared for on a daily basis.

Regarding the knowledge sharing medium, the participants tended to comment on the messages of others more than starting new conversations which indicates that, as in previous research, simply reading information from other caregivers may have a therapeutic effect [42]. The label mainly used in the messages of the two communities was “caring for others”. Therefore, they were more concerned about acquiring knowledge related to caring for the people with Alzheimer’s rather than with caring for themselves, probably due to their strongly developed sense of responsibility and duty towards their relatives [16]. The purpose of the messages was to give information about the sender, not seek answers. It seems that the interaction was more to socialise and to feel heard.

Regarding the knowledge sharing context, both communities appear to have experienced a positive impact of the face-to-face meetings. Studies suggest that face-to-face meetings contribute to the strengthening of the bonds between members of primarily virtual groups [43]. In the early stages in particular, trust needs to be built and higher-level functions such as information sharing, knowledge sharing and creation come later. The community exclusively with caregivers achieved a level of trust first, perhaps because they had more focus on socialising between peers than counting on health professionals. With regard to the topics of information shared, the influence of health professionals is reflected in the “I’mWithYouPlus” virtual community of practice where the informative messages were split between the sender and disease, sharing more explicit and experiential knowledge.

As the community grows, it develops a systematic body of knowledge [44]. Each community had its own temporal pattern that in some way mirrored the pattern of the practice (practitioners probably were busy in the morning but could spare more time in the evening). The advantage of virtuality offering 24-hour community availability allowed participants to use it at their own convenience.

The dominant form of sharing was social support. As in previous studies [45, 46], participants shared requests for support, offered support and gratitude for help received. It is plausible to suggest that they benefited from the feeling of connectivity to others in similar situations as a high level of social support is related to a lower burden on the caregiver [47]. However, this focus on social support, which seems to be more valued by the caregivers than information about the disease, was not an expected pattern as one of the most highlighted caregivers’ needs is to have access to information [24]. Furthermore, in neither community was sharing knowledge about care for the caregiver very important. This might be seen as rather surprising but reflects that the community offered support itself, rather than discussion of how to gain support. There was also some sharing of knowledge about the project itself.

In terms of the knowledge sharing modality, even though communities of practice offer a framework for sharing experiential knowledge [48], caregivers adapted the virtual community of practice to their own needs and shared mainly social knowledge. In terms of social exchange, members are willing to provide valuable information and share with other members. Our analysis found that many users come to the site solely to express their emotions and seek affirmation from fellow caregivers. The developers of the LINKS method were preoccupied with the turning of valuable but intangible expert doctor knowledge from experiential to explicit knowledge [30]. Their framework of analysis was very helpful to this study, but the pattern of knowledge sharing in our case was very different. Social knowledge rather than expert knowledge was the focus of knowledge sharing.

This study reflects that the communities were a support community, and that emotional support is the principal need of caregivers, as discussed in the literature review. There were also relevant findings about when messaging occurred which emphasises the importance of the moderators’ profile [7]. This study corroborates this statement as the virtual community of practice, including health professionals as moderators, behaves differently from a virtual community of practice without health professionals. Virtual communities of practice where peers count on each other, function more as a support group, whereas those moderated by health professionals’ function more as a place to go to acquire information. Therefore, the selection of the moderators in these virtual communities of practice for caregivers of people with Alzheimer’s should be tailored to the aim of the intervention.

### Limitations

The caregivers’ age, together with their eHealth literacy, excluded a lot of caregivers from the research. Moreover, a specific study of the moderators’ participation could have helped highlight more detailed results regarding their role in the communities. Finally, with regards to lurkers, who read messages but did not post many, it would have been interesting to have had full traceability of them in via app in order to obtain more details about the exchange of knowledge.

## Conclusions

Caregivers of people with Alzheimer’s need to learn skills for improving the daily life management of their relative, to have access and guidance to information, and to receive social support. This research emphasises the importance of social knowledge sharing and social support to caregiver communities. It shows that supportive relationships are found within the virtual communities of practice and that caregivers of people with Alzheimer’s use these communities as an escape valve. Trust is the glue that binds and encourages the members of a community to act in a sharing and flexible manner. Caregivers feel comfortable in this sort of community where they can gain social recognition from their peers. Therefore, the benefit of virtual communities of practice lies not in the content but in the ongoing social interaction which is rooted in the reality of community.

This study contributes to use of the LINKS model as a framework to this specific context because it demonstrates its value, and usefully extends this with a validated set of content categories for knowledge sharing. Moreover, this research stresses the importance of social knowledge sharing and social support to caregiver communities. Caregivers of people with Alzheimer’s need to learn skills for improving the daily life management of their relative, to have access and guidance to information, and to receive social support. This study shows that supportive relationships are found within the virtual communities of practice.

The paper has contributed insights into the nature of knowledge sharing in support communities and how the character of this is subtly different when practitioners are or are not present. The level of interaction points to this being a forum for knowledge sharing, rather than mere knowledge transfer. Even though many studies suggested more information should be provided to caregivers, with some providing topical areas in which information might be useful, we have seen that caregivers are desperate for social support. It reinforces the value of recognising that interactive knowledge sharing in a community has its place alongside knowledge transfer from expert practitioners to caregivers. Therefore, healthcare delivery models should take into account these kinds of internet-based intervention, aimed at supporting caregivers. The health system should take advantage of its potential.

## Data Availability

The datasets used and/or analysed during the current study available from the corresponding author on reasonable request.

## References

[1] Hafeez K, Abdelmeguid H (2003). Dynamics of human resource and knowledge management. J Oper Res Soc.

[2] Lave J, Wenger E. Situated learning: legitimate peripheral participation. Cambridge University Press; 1991.

[3] Vidgen R, Sims JM, Powell P (2013). Do CEO bloggers build community?. J Commun Manag.

[4] Jagasia J, Baul U, Mallik D (2015). A Framework for communities of Practice in Learning organizations. Bus Perspect Res.

[5] Lesser EL, Storck J (2001). Communities of practice and organizational performance. IBM Syst J.

[6] Sibte S, Abidi R. Healthcare Knowledge Sharing: Purpose, Practices, and Prospects. In: Bali, R.K., Dwivedi. A., eds. *Healthcare Knowledge Management: Issues, Advances and Successes*. Springe. Springer New York LLC; 2006:67–86.

[7] Ranmuthugala G, Plumb J, Cunningham F, Georgiou A, Westbrook J, Braithwaite J. How and why are communities of practice established in the healthcare sector? A systematic review of the literature. BMC Heal Serv Res. 2011;11. 10.1186/1472-6963-11-273. 10.1186/1472-6963-11-273 PMC3219728 21999305

[8] Fulk J, Flanagin AJ, Kalman ME, Monge PR, Ryan T (1996). Connective and communal public goods in interactive communication systems. Commun Theory.

[9] Jiménez-Zarco AI, González-González I, Saigí-Rubió F, Torrent-Sellens J (2015). The co-learning process in healthcare professionals: assessing user satisfaction in virtual communities of practice. Comput Hum Behav.

[10] Zhao Y, Feng H, Hu M, et al. Web-based interventions to Improve Mental Health in Home caregivers of people with dementia: Meta-Analysis. JMIR. 2019;2(5). 10.2196/13415. 10.2196/13415 PMC6526687 31066680

[11] Pleasant M, Molinari V, Dobbs D, Meng H, Hyer K (2020). Effectiveness of online dementia caregivers training programs: a systematic review. Geriatr Nurs (Minneap).

[12] Ministerio de Sanidad - Consumo y Bienestar. *Plan Integral de Alzheimer y Otras Demencias (2019–2023)*.; 2019.

[13] Crous-Bou M, Minguillón C, Gramunt N, Molinuevo JL (2017). Alzheimer’s disease prevention: from risk factors to early intervention. Alzheimer’s Res Ther.

[14] Jeste DV, Mausbach B, Lee EE (2021). Caring for caregivers/care partners of persons with dementia. Int Psychogeriatr.

[15] Plöthner M, Schmidt K, De Jong L, Zeidler J, Damm K (2019). Needs and preferences of informal caregivers regarding outpatient care for the elderly: a systematic literature review. BMC Geriatr.

[16] Masana L (2017). Cuidados informales de larga duración en España: Retos, miradas y soluciones. Salud Colect.

[17] Wolff JL, Roter DL (2008). Hidden in plain sight: medical visit companions as a Resoursce for vulnerable older adults. Arch Intern Med.

[18] Gillick MR (2013). The critical role of caregivers in achieving patient-centered care. JAMA.

[19] Wu KC, Su Y, Chu F, Chen AT, Zaslavsky O. Behavioral change factors and Retention in web-based interventions for Informal caregivers of people living with dementia: scoping review. J Med Internet Res. 2022;24(7). 10.2196/38595. 10.2196/38595 PMC9305400 35797100

[20] Chiu T, Marziali E, Colantonio A (2009). Internet-based caregiver support for Chinese canadians taking care of a family member with alzheimer disease and related dementia. Can J Aging.

[21] Ferrucci F, Jorio M, Marci S (2021). A web-based application for complex health care populations: user-centered design approach. JMIR Hum Factors.

[22] Reinhard S, Young HM, Levine C, Kelly K, Choula RB, Accius JC. *Home Alone Revisited: Family Caregivers Providing Complex Care*. Vol 3.; 2019. 10.1093/geroni/igz038.2740.

[23] Ashrafizadeh H, Gheibizadeh M, Rassouli M, Hajibabaee F, Rostami S (2021). Explain the experience of family caregivers regarding care of Alzheimer’s patients: a qualitative study. Front Psychol.

[24] Queluz FNFR, Kervin E, Wozney L, Fancey P, Mcgrath PJ, Keefe J (2020). Understanding the needs of caregivers of persons with dementia: a scoping review. Int Psychogeriatr.

[25] Swartz K, Collins LG (2019). Caregiver care. Am Fam Physician.

[26] Lee SM, Lee Y, Choi SH, Lim TS, Moon SY (2019). Clinical and demographic predictors of adverse outcomes in caregivers of patients with dementia. Dement Neurocognitive Disord.

[27] Subramaniam A, Mehta KK. Exploring the lived experiences of Caregiving for Older Family members by Young caregivers in Singapore: transition, trials, and tribulations. Int J Environ Res Public Health. 2024;21(2). 10.3390/ijerph21020182. 10.3390/ijerph21020182 PMC10888348 38397673

[28] Wangler J, Jansky M (2021). Support, needs and expectations of family caregivers regarding general practitioners – results from an online survey. BMC Fam Pract.

[29] Whitlatch CJ, Orsulic-jeras S, Rose B (2017). Meeting the Informational, Educational, and Psychosocial Support needs of persons living with dementia and their family. Gerontologist.

[30] Abidi SSR, Hussini S, Sriraj W, Thienthong S, Finley GA (2009). Knowledge sharing for pediatric pain management via a web 2.0 framework. Stud Health Technol Inf.

[31] Xu WW, Chiu IH, Chen Y, Mukherjee T. Twitter hashtags for health: Applying network and content analyses to understand the health knowledge sharing in a Twitter-based community of practice. *Qual Quant Interntational Journl Methodol*. Published online 2014.

[32] McAdam R, McCreedy S (2000). A critique of knowledge management: using a social constructionist model. New Technol Work Employ.

[33] Stewart SA, Abidi SSR. Applying social network analysis to understand the knowledge sharing behaviour of practitioners in a clinical online discussion forum. J Med Internet Res. 2012;14(6). 10.2196/jmir.1982. 10.2196/jmir.1982 PMC3799555 23211783

[34] Gaugler JE, Westra BL, Kane RL. Professional discipline and support recommendations for family caregivers of persons with dementia. *Int Psychogeriatr*. 2016;28(6):1029–1040. doi:110.1016/j.bbi.2017.04.008. 10.1017/S1041610215002318 PMC5767315 26739379

[35] Boada DA. Cultivating an online teacher community of practice around the Instructional Conversation Pedagogy: a Social Network Analysis. Volume 70. Springer US; 2022. 10.1007/s11423-021-10058-9.

[36] Boots LMM, De Vugt ME, Van Knippenberg RJM, Kempen GIJM, Verhey FRJ (2014). A systematic review of internet-based supportive interventions for caregivers of patients with dementia. Int J Geriatr Psychiatry.

[37] Wenger E, Trayner B. Why focus on communities of practice. Published 2021. Accessed February 26, 2021. https://wenger-trayner.com/project/why-focus-on-communities-of-practice/.

[38] Romero-Mas M, Gómez-Zúñiga B, Cox AM, Ramon-Aribau A. Designing virtual communities of practice for informal caregivers of Alzheimer’s patients: an integrative review. Health Inf J. 2020;26(4). 10.1177/1460458220950883. 10.1177/1460458220950883 32951497

[39] Waters RD, Burnett E, Lamm A, Lucas J (2009). Engaging stakeholders through social networking: how nonprofit organizations are using Facebook. Public Relat Rev.

[40] Michon A, Canuto A, Giardini U, Giannakopoulos P, Weber K, Gargiulo M (2004). Caregiver burden in dementia: determinants and interventions. Schweizer Arch fur Neurol Und Psychiatr.

[41] Adelman RD, Tmanova LL, Delgado D, Dion S, Lachs MS (2014). Caregiver burden: a clinical review. JAMA - J Am Med Assoc.

[42] Whitlatch CJ, Orsulic-jeras S, Rose B (2018). Meeting the Informational, Educational, and Psychosocial Support needs of persons living with dementia and their family caregivers. Gerontologist.

[43] Salido MJ. *Comunidades de Práctica: Una Metodología Para Construir, Desarrolar y Fortalecer Redes de Conocimiento*. 1st. (The Project Working on Solution SL, ed.).; 2012.

[44] Wenger MDR, Dydnew W (2002). Cultivating communities of Practice, A Guide to managing knowledge.

[45] Hopwood J, Walker N, McDonagh L, et al. Internet-based interventions aimed at supporting family caregivers of people with dementia: systematic review. J Med Internet Res. 2018;20(6). 10.2196/jmir.9548. 10.2196/jmir.9548 PMC6019848 29895512

[46] Scharett E, Lopes S, Rogers H (2017). An investigation of information sought by caregivers of Alzheimer’s patients on online peer-support groups. Proc Hum Factors Ergon Soc.

[47] Wang Z, Ma C, Han H (2018). Caregiver burden in Alzheimer’s disease: moderation effects of social support and mediation effects of positive aspects of caregiving. Int J Geriatr Psychiatry.

[48] Chen Z, Shepherd M, Sibte S, Abidi R, Finley GA. Linking Tacit Knowledge in the Pediatric Pain e-Mail Archives and Explicit Knowledge in PubMed. In: *Proceedings of the 39th Hawaii International Conference on System Sciences*. Vol 00.; 2006:1–10.

